# Antibiotic Use in Pediatric Dengue Cases in Taiwan: A National Administrative Database Analysis

**DOI:** 10.4269/ajtmh.24-0414

**Published:** 2025-05-27

**Authors:** Yi-Jung Shen, Chia-En Lien, Yiing-Jenq Chou, Nicole Huang, Theodore Tsai

**Affiliations:** ^1^ Institute of Hospital and Health Care Administration, College of Medicine, National Yang Ming Chiao Tung University, Taipei, Taiwan;; ^2^ Department of Health Law, Policy, and Management, School of Public Health, Boston University, Boston, Massachusetts;; ^3^ Institute of Public Health, College of Medicine, National Yang Ming Chiao Tung University, Taipei, Taiwan;; ^4^ Office of the Deputy Superintendent, National Yang Ming Chiao Tung University Hospital, Yilan County, Taiwan;; ^5^ Takeda Vaccines Inc., Cambridge, Massachusetts

## Abstract

Nonspecific dengue clinical manifestations lead to avoidable antibiotic use. We describe antibiotic use in confirmed pediatric dengue cases and associated healthcare utilization and medical costs, including from antibiotic-associated adverse drug events (ADEs). From Taiwanese national administrative databases, children with confirmed dengue between 2009 and 2015 who were younger than 18 years old, without bacterial infection, and who were prescribed antibiotics a week before or after laboratory diagnosis were propensity score matched and compared with nonrecipients, using regression models for ADEs and medical care expenditures. A total of 1,959 out of 6,938 children (28.2%) with confirmed dengue without bacterial infection received antibiotics. Recipients were more likely to incur emergency department visits (odds ratio [OR]: 3.16; 95% CI: 2.62–3.81), hospitalizations (OR: 4.20; 95% CI: 3.48–5.07), and higher predicted medical care expenditures (new Taiwan dollar [NTD] $16,914 versus NTD $14,104) in a 30-day follow-up period. They also had significantly higher odds of overall defined (OR: 1.72; 95% CI: 1.42–2.09), dermatologic-related (OR: 1.83; 95% CI: 1.36–2.46), and gastrointestinal-related (OR: 1.67; 95% CI: 1.31–2.12) ADEs. A substantial proportion of pediatric patients with dengue received potentially avoidable courses of antibiotics. Recipients were more likely to experience ADEs, incurring more medical care utilization and expenditures than untreated cases.

## INTRODUCTION

Inappropriate antibiotic prescribing in healthcare is a major contributor to antibiotic resistance, a global public health threat.^1^^–^^3^ The frequency of inappropriate antibiotic use is highest in low- to middle-income countries.^3^^–^^5^ Dengue, a mosquito-borne flaviviral infection, is endemic throughout tropical and subtropical regions in more than 100 countries, principally affecting low- to middle-income populations.^6^ The overlapping nonspecific clinical manifestations of dengue virus infection with other febrile illnesses in these locations, including bacterial infections such as leptospirosis for which antibiotic therapy is appropriate, increases the potential for antibiotic misuse and overuse.^6^^,^^7^ Respiratory infections are the focus of most studies on antibiotic misuse, and few studies of antibiotic use for dengue have been reported. A review of case series conducted under diverse circumstances and in various patient populations reported that 15–75% of patients with dengue received a course of antibiotics,^8^ but we are unaware of any large-scale population-based studies of antibiotic use in pediatric dengue cases.

Antibiotics are responsible for more emergency department (ED) visits for adverse events after therapy than any other category of drugs, even after accounting for higher prescribing rates for antibiotics.^9^ We previously explored the nationwide infectious disease-reporting system in Taiwan and patient-level claims data to assess patterns of antibiotic prescription and medical expenditures among laboratory-confirmed adult dengue cases.^10^ Whereas children are susceptible to dengue and potentially inappropriate antibiotic prescribing, there have been no reports of adverse events and medical spending associated with prescribing antibiotics among confirmed pediatric dengue cases.

Here, we describe antibiotic use in pediatric patients with confirmed dengue, including the occurrence of potentially related adverse events associated with antibiotic use and their costs, following a previously reported paradigm for antibiotic-related adverse events in children with viral respiratory infection or otitis media.^11^

## MATERIALS AND METHODS

### Data source and study sample.

This study was reviewed and approved by the Institutional Review Board of National Yang Ming Chiao Tung University (Noet needed YM109083E). We used the Notifiable Disease Dataset of Confirmed Cases, maintained and provided by the Taiwan Centers for Disease Control (TCDC), to identify laboratory-confirmed dengue cases among children younger than 18 years of age, and their date of confirmed diagnosis.^10^ Under the Communicable Disease Control Act, all physicians and healthcare facilities are required to submit specimens from suspected dengue cases to the TCDC. Confirmed dengue cases were linked to the National Health Insurance (NHI) Research Database (NHIRD) to obtain information on demographics, healthcare utilization, and spending on medical care for the children identified. Medical encounters must be reported to the NHIRD for reimbursement; therefore, compliance is high. All analyses were conducted onsite at the Health and Welfare Data Center.

A total of 8,095 children younger than 18 years with confirmed dengue between January 1, 2009, and December 31, 2015, were identified. We excluded children with multiple confirmed dengue records within 3 months from the initial dengue confirmation date, without complete demographic characteristics, having no medical care encounters, or having bacterial infection diagnoses within 7 days before and after the date of confirmed dengue diagnosis. The remaining sample included 6,938 laboratory-confirmed pediatric dengue cases without a reported secondary bacterial infection.

### Identification of antibiotic prescribing.

Each child with confirmed dengue was assessed for any antibiotic prescribed within the 7 days before or after the date of confirmed dengue infection. We adopted the Anatomical Therapeutic Chemical (ATC) classification system to identify the type of antibiotic prescribed (the first three digits of the ATC code in J01). Children who had at least one antibiotic prescribed during the 14-day assessment interval were defined as the treatment group (*n* = 1,959). Those who did not have an antibiotic prescribed during the 14-day interval comprised the comparison group (*n* = 4,979).

To reduce possible confounding by indication between treatment and comparison groups, we used the propensity score matching (PSM) method with one to one matching using a greedy algorithm.^12^ Hence, the post-PSM sample included the treatment group (*n* = 1,959) and the comparison group (*n* = 1,959). The post-PSM sample was used for the main analyses. The variables used to calculate propensity score matching included age, gender, parental socioeconomic status (SES), and health status, confirmed during the “epidemic period” or not. Age was classified into five groups: younger than 1, 1–4, 5–9, 10–14, and 15–17 years. The health status variable was constructed as a dichotomous variable indicating whether the patient had a major comorbid condition as listed in the Catastrophic Illness list maintained by the NHI Administration, such as rare disease, cancer, or chronic kidney disease. Parental SES was defined by the primary insurance holder’s insurable wages and types. Individuals with a well-defined monthly wage were classified into two categories: less than new Taiwan dollar (NTD) $30,000 and NTD $30,000 or more (NTD $30 ≈ United States dollar [USD] $1). A small number without well-defined monthly wages was identified separately.

### Outcome variables.

The main outcome variables were adverse events associated with antibiotics and spending on medical care. To assess adverse drug events (ADEs) associated with prescribing antibiotics for dengue, we adopted the definitions and algorithms developed by Butler et al. to identify antibiotic-related ADEs during various follow-up periods using *International Classification of Diseases, Ninth and Tenth Revisions, Clinical Modification* diagnosis codes (see Supplemental Table 1 for more specific definitions and assessment algorithms).^11^ For children who received antibiotics, the index date was defined as whichever date came first—antibiotic initiation or diagnosis for antibiotic group; for those who did not receive an antibiotic prescription, the date of their confirmed dengue diagnosis. The follow-up periods ranged from 2 to 90 days after the index date. ADEs were collected and were stratified into dermatologic- and gastrointestinal-related events, per Butler et al.^11^ Individual ADEs were grouped into larger categories because the NHIRD regulations prohibit analyses of any cell for which the number of events is between one and three. We also followed each child for any occurrence of ED visits and hospitalization 14 and 30 days after the index date. The presence of any ADEs, ED visits, and hospitalizations were constructed as binary variables. Total medical care costs (including outpatient, emergency, and inpatient settings) incurred within 30 days after the index date under the NHI program were assessed. The cost variables were constructed as continuous variables.

## STATISTICAL ANALYSES

Descriptive statistics were computed to describe the rates of antibiotic prescriptions and adverse events, the mean medical care expenditure, and the characteristics of children with dengue. Multiple logistic regression models were applied to assess the association between prescribing antibiotics, ADEs, and occurrence of emergency and inpatient service use. Because the medical care costs were skewed, multivariable linear regression models with log transformation of medical care costs were applied to assess the association between prescribing antibiotics and medical care expenditure among children with dengue. Patient characteristics and the index year were included as covariates in the models. We conducted two sensitivity analyses: 1) to exclude patients who had been hospitalized before the dengue confirmation date, and 2) to apply a Cox proportional hazards model to determine hazard ratios of adverse events. All analyses were conducted using SAS 9.4 (SAS Institute, Cary, NC).

## RESULTS

Of 6,938 children with dengue, 1,959 children (28.2%) were prescribed an antibiotic within 7 days before or after their dengue infection was confirmed, but 4,979 children (71.8%) were not (Table 1). Children between 15 and 17 years old had higher rates of antibiotic receipt (30.6%) than younger patients (25.5%). A greater proportion of children with preexisting major comorbid conditions were prescribed antibiotics (36.4% versus 28.1% in those without those conditions; *P* = 0.030). Antibiotics were prescribed less often to cases during the two major epidemic years (2014 and 2015) than in other years (27.7% versus 34.3%, respectively; *P* <0.001). After propensity score matching, there were no significant differences between the children who had or did not have an antibiotic prescription in all matching variables (Table 1).

**Table 1:** Characteristics of children with dengue for pre- and post-propensity score matching (PSM)

Characteristic	Before PSM				After PSM		
	Antibiotic, *n*	Total, *n*	%	*P*-Value	Antibiotic, *n* (%)	No Antibiotic, *n* (%)	*P*-Value
Total cases	1,959	6,938	28.2	–	1,959	1,959	–
Child’s age, years				0.064			1.000
<1	36	105	25.5		36 (1.8)	35 (1.8)	
1–4	169	439	27.8		169 (8.6)	170 (8.7)	
5–9	445	1,133	28.2		445 (22.7)	440 (22.5)	
10–14	704	1,929	26.7		704 (35.9)	708 (36.1)	
15–17	605	1,373	30.6		605 (30.9)	606 (30.9)	
Child’s gender				0.638			0.872
Male	1,121	2,880	28.0		1,121 (57.2)	1,116 (57.0)	
Female	838	2,099	28.5		838 (42.8)	843 (43.0)	
Parental SES, NTD$				0.862			0.988
<$30,000	866	2,168	28.5		866 (44.2)	867 (44.3)	
≥$30,000	596	1,522	28.1		596 (30.4)	599 (30.6)	
Others	497	1,289	27.8		497 (25.4)	493 (25.2)	
Major comorbid conditions				0.030			0.920
No	1,908	4,890	28.1		1,908 (97.4)	1,909 (97.4)	
Yes	51	89	36.4		51 (2.6)	50 (2.6)	
Epidemic period*				<0.001			0.959
No	207	396	34.3		207 (10.6)	208 (10.6)	
Yes	1,752	4,583	27.7		1,752 (89.4)	1,751 (89.4)	

NTD = new Taiwan dollar, United States dollar $1 ≈ NTD $30; PSM = propensity score matching; SES = socioeconomic status.

* Epidemic period was 2014–2015.

With respect to antibiotic-associated ADEs, children who had been prescribed antibiotics had higher rates of all prespecified ADEs (15.8% versus 9.8%; *P* <0.001), dermatologic-related ADEs (6.4% versus 3.6%; *P* <0.001), and gastrointestinal-related ADEs (9.6% versus 6.0%; *P* <0.001). They also were more likely (approximately three times) to incur ED visits in either 14-day (22.9% versus 8.3%; *P* <0.001) or 30-day (23.7% versus 9.5%; *P* <0.001) follow-up periods, and hospitalization in either 14-day (27.2% versus 7.9%; *P* <0.001) or 30-day (27.7% versus 8.4%; *P* <0.001) follow-up periods compared with their counterparts. The average medical care expenditure for children with dengue who were prescribed antibiotics was NTD $11,017, almost double the costs among those who did not receive an antibiotic (NTD $6,113) (Table 2).

**Table 2:** Adverse events and medical care costs in post-PSM patients with dengue who were prescribed at least one antibiotic and those did not prescribe any antibiotic

Event	Antibiotic (*N* = 1,959)	No Antibiotic (*N* = 1,959)	*P*-Value		Antibiotic Versus No Antibiotic	
	*n* (%)	*n* (%)			OR	95% CI
Adverse drug events						
All	309 (15.8)	192 (9.8)	<0.001		1.72	1.42–2.09
Dermatologic	126 (6.4)	71 (3.6)	<0.001		1.83	1.36–2.46
Gastrointestinal	189 (9.6)	118 (6.0)	<0.001		1.67	1.31–2.12
ED visit*						
14 days	448 (22.9)	163 (8.3)	<0.001		3.45	2.84–4.20
30 days	464 (23.7)	186 (9.5)	<0.001		3.16	2.62–3.81
Hospitalization*						
14 days	533 (27.2)	154 (7.9)	<0.001		4.19	3.46–5.07
30 days	542 (27.7)	164 (8.4)	<0.001		4.20	3.48–5.07
	Mean (IQR)	Mean (IQR)	*P*-Value		Predicted value, NTD$	95% CI
Total medical care expenditure, NTD$	11,017 (2,702–14,653)	6,113 (1,396–8,147)	<0.001	Antibiotic	16,914.81	14,632.20–19,197.41
				Nonantibiotic	14,104.14	11,819.63–16,388.64

ED = emergency department; IQR = interquartile range; NTD = New Taiwan Dollar, United States dollar $1 ≈ NTD $30; OR = odds ratio. All adverse drug events included skin rash, Stevens-Johnson syndrome, toxic epidermal necrolysis, urticaria, nausea/vomiting, abdominal pain, non–*Clostridioides *(*C.*)* difficile* diarrhea, *C. difficile* infection, anaphylaxis, angioedema, laryngeal edema, and unspecified allergy. Dermatologic-related drug events included skin rash, Stevens-Johnson syndrome, toxic epidermal necrolysis, and urticaria. Gastrointestinal-related drug events included nausea/vomiting, abdominal pain, non–*C. difficile* diarrhea, and *C. difficile* infection.

* Indicates days after antibiotic prescription or, in comparison group, days after dengue diagnosis.

Children who were prescribed antibiotics were significantly more likely to experience overall ADEs (odds ratio [OR]: 1.72; 95% CI: 1.42–2.09), dermatologic-related ADEs (OR: 1.83; 95% CI: 1.36–2.46), and gastrointestinal-related ADEs (OR: 1.67; 95% CI: 1.31–2.12). Children who were prescribed antibiotics were 3.45 times (95%; CI: 2.84–4.20) and 4.19 times (95% CI: 3.46–5.07) more likely to incur an ED visit and hospitalization, respectively, during the 14-day follow-up period. Similar findings were found for a longer 30-day follow-up period. Children who were prescribed antibiotics were 3.16 times (95% CI: 2.62–3.81) and 4.20 times (95% CI: 3.48–5.07) more likely to visit an ED and be hospitalized, respectively, during the 30-day follow-up period. The total medical care expenditure of children who were prescribed antibiotics was 180% higher than the total medical expenditure of untreated children. After adjustment, the predicted medical care expenditure for children who were prescribed antibiotics was NTD $16,914.81, whereas the predicted expenditure of those who were not prescribed antibiotics was NTD $14,104.14 (Table 2).

Sensitivity analyses were conducted by excluding children with dengue who were admitted before their dengue infections were confirmed. The main results remained robust after we applied a sensitivity analysis.

## DISCUSSION

This population-based study in Taiwan investigated antibiotic-related adverse events in children with dengue, and associated healthcare utilization and medical care expenditures among the treated cases compared with untreated cases. In principle, antibiotic therapy for this viral infection is unnecessary; therefore, antibiotic-related ADEs and associated healthcare utilization and their costs also are potentially avoidable if dengue is prevented. To describe these outcomes, we compared antibiotic-treated patients versus untreated patients with dengue (excluding patients with secondary bacterial infection) by linking data in national databases that cover 99% of the Taiwanese population with all reported dengue cases over an 8-year interval. Because adverse events associated with antibiotic use are common and should be accounted for in an assessment of the impact of unnecessary antibiotic use, we also captured specified antibiotic-treatment ADEs to provide a comprehensive analysis of antibiotic-associated healthcare utilization and their costs. With the high compliance for reporting to these national databases, the results effectively provide an ascertainment of these potentially avoidable outcomes in Taiwan.

The significantly higher risks of prespecified ADEs among antibiotic-treated dengue cases represent morbidity that was avoidable because of unneeded antibiotic use in these confirmed infections of viral etiology. Consistent with previous studies of inappropriate antibiotic prescriptions for viral infections, we found a higher risk of prespecified dermatologic and gastrointestinal ADEs that are frequent side effects of antibiotic therapy.^11^ Interestingly, broad-spectrum cephalosporins were the most frequently prescribed among all antibiotics (Supplemental Table 2). Cephalosporins may be used empirically in cases of acute febrile illness without clear etiology, and with initial symptoms that are nonspecific, individual dengue cases and even outbreaks have been attributed initially to leptospirosis or typhus, as well as acute viral infection, leading to precautionary antibiotic prescribing.^13^^–^^15^ We were unable to describe the frequency of rare but potentially life-threatening adverse events, such as anaphylaxis, Stevens-Johnson syndrome, toxic epidermal, or *Clostridioides difficile* infection because of limitations imposed by the NHIRD.

Antibiotic-treated dengue cases experienced a three- to four-times higher likelihood of ED visits and hospitalization over 14- and 30-day follow-up intervals: one in five children with an antibiotic prescription had at least one ED visit, and one in four had at least one hospital admission within 30 days after the index date. The ED visits and hospitalizations occurred mostly within 14 days after the index date for both groups. This healthcare utilization led to 1.8 times higher total medical care expenditure within the 30-day interval in antibiotic-treated dengue compared with untreated cases. After adjusting for demographics, SES, and health status, overall predicted medical expenditure among treated cases was NTD $16,914.81, which was 20% higher in those not receiving antibiotics (NTD $14,104.14). The contribution of antibiotic cost itself to this difference is minor because the average value point of antibiotics per capsule in the NHI program is NTD $117.33, and more than 90% are less than NTD $300, which is approximately USD $10.

The higher frequency of antibiotic prescription for patients with comorbidities than in healthy children (36.4% versus 28.1%, respectively; *P* = 0.030) supports the propensity matching that was undertaken to adjust for underlying risks that may have influenced dengue severity. Although we excluded patients with a bacterial infection, we cannot exclude that antibiotic-treated patients presented with a more severe clinical presentation that let to ED visits, hospitalizations, and precautionary antibiotic prescription. In the context of an administrative database study, we were limited in our ability to describe and adjust for the acuity of illness in the two groups. However, as antibiotics are implicated in more outpatient visits for treatment-related adverse events than any other class of drug, our findings of increased healthcare utilization and medical costs in treated children are consistent with previous experience.^11^^,^^16^^,^^17^

Another result from this study that warrants attention is the lower use of antibiotics among dengue cases during an unusual 2-year period of epidemic transmission in 2014–2015 (27.7% versus 34.3% in other years). The number of reported dengue cases in those 2 years was two to three orders of magnitude greater than in other years. We speculate that clinicians, being aware during that interval that dengue was a likely diagnosis among children presenting with an undifferentiated febrile illness, were more likely to refrain from prescribing an antibiotic.

Overall, the study provides a valuable insight into the avoidable burden of antibiotic use for dengue, a disease with near-global reach that continues to increase in scale. As an acute undifferentiated febrile illness, it is understandable that some patients will be prescribed antibiotics presumptively. Point-of-care tests for dengue were not available in Taiwan until 2015 and their performance and availability are still limited. Even if improved point-of-care tests are developed, access in low- to middle-income and developing countries where dengue is endemic will need to be addressed. However, if dengue was prevented or reduced in the first place, by improved sanitation, vector control, and immunization, those cases, as well as subsequent antibiotic use, would be prevented or reduced.

If the proportion of cases treated with antibiotics in Taiwan (28.2%) held true for other countries and regions, tens of millions of antibiotic courses could be averted if dengue was prevented. However, it is likely that this is a conservative estimate because antibiotic out-of-pocket purchases would not have been captured by our analysis of the NHI scheme. Antibiotics may be even more readily available in other countries where prescriptions are not required and where dengue is endemic. In low-income countries, inappropriate antibiotic treatment of children is well recognized as problematic, with a remarkable 83% of all pediatric outpatients having received antibiotics in a Cambodian study and 97% of children presenting with acute respiratory illness in a study in Vietnam.^4^^,^^5^ The additional morbidity and cost burdens associated with antibiotic-associated ADEs should be included in assessments of the global disability-adjusted life-years and economic burdens of dengue.

There are several limitations to this study. Antibiotic usage not captured through the health insurance database analysis from, for example, out-of-pocket purchases and the use of leftover or borrowed antibiotics and associated ADEs would not have been accessible. Furthermore, the NHI outpatient claims data only record up to three diagnoses, resulting in potential underestimation of ADEs. Cephalosporins were the most frequently prescribed class of antibiotics in this experience and broad-spectrum antibiotics are more likely to be associated with ADEs than narrow-spectrum antibiotics; as antibiotic availability and use practices in other countries may differ, the frequency of treatment-associated ADEs also might differ.^18^ We only linked NHI claims data to calculate the medical care costs among children with dengue; nonmedical costs resulting in economic burden were not estimated. Beyond the acute adverse impacts of antibiotic therapy, disturbances of the gut microbiome and propagation of antibiotic-resistant organisms, as well as broader health impacts across the life course (e.g., asthma), have been described, and describing these longer-term impacts were beyond the scope of this study.^19^^–^^22^ Most importantly, because we did not review individual medical records, we could not discriminate the severity of individual cases that might have influenced the frequency of ED visits and hospitalizations and their associated costs. This study was conducted under an NHI program, with high accessibility to healthcare services, and so the results might not be generalizable to other healthcare delivery systems.

## CONCLUSION

This population-based study is the first to investigate antibiotic administration in confirmed pediatric dengue cases and subsequent antibiotic-associated ADEs, healthcare utilization, and costs. Antibiotics were prescribed in nearly one-third of confirmed dengue cases that did not include a secondary bacterial infection. Incidence of ADEs, healthcare utilization, and costs were greater in children who received an antibiotic than in untreated children, but differences in the acuity of illness that also may have contributed to these outcomes could not be ruled out. The investigation also calls attention to an important gap in antibiotic stewardship efforts in Taiwan and other locations where dengue is endemic.

## Supplemental Materials

10.4269/ajtmh.24-0414 Supplemental Materials
