# Supplementary material for: Antibiotic Use in Pediatric Dengue Cases in Taiwan: A National Administrative Database Analysis
Source: Am J Trop Med Hyg. 2025 May 27;113(2):347–52. doi: 10.4269/ajtmh.24-0414 (PMC12360090; doi:10.4269/ajtmh.24-0414)
Supplement: Supplemental Materials [file tpmd240414.SD1.pdf]

SUPPLEMENTARY TABLE S1

Codes and timing to identify adverse drug events associated with antibiotic prescription for dengue

Adverse drug event	Follow-up duration from the index date	ICD-10-CM codes	ICD-9-CM codes
Dermatologic			
Skin rash	1–14 days	R21	782.1
Stevens-Johnson syndrome	1–14 days	L51.1	695.13
Toxic epidermal necrolysis	1–14 days	L51.2–L51.3	695.14, 695.15
Urticaria	1–14 days	L50.0–L50.1, L50.6–L50.9	708
Gastrointestinal			
Nausea/vomiting	1–14 days	R11.0–R11.12, R11.2	787.0
Abdominal pain	1–14 days	R10.0–R10.33, R10.84–R10.9	789.0
Non-*Clostridioides difficile* diarrhea	1–30 days	K52.29–K52.3, K52.831–K52.9, R19.7	558.3, 558.9, 787.91
C. difficile infection	1–90 days	A04.7–A04.72	008.45

Hypersensitivity			
Anaphylaxis	0–2 days	T78.2XXA, T88.6XXA	995.0
Angioedema	0–2 days	T78.3XXA	995.1
Laryngeal edema	0–2 days	J38.4	478.6
Unspecified allergy	1–14 days	T78.40XA, T78.49XA	995.3

ICD-9/10-CM = *International Classification of Diseases, Ninth and Tenth Revisions, Clinical Modification*.

All adverse drug events included Skin rash, Stevens-Johnson syndrome, Toxic epidermal necrolysis, Urticaria, Nausea/vomiting, Abdominal pain, Non-*C. difficile* diarrhea, *C. difficile* infection, Anaphylaxis, Angioedema, Laryngeal edema, and Unspecified allergy.

SUPPLEMENTARY TABLE S2

The top 5 most commonly prescribed antibiotics in confirmed dengue cases

No.	Antibiotics	*N*
1	Amoxicillin	618
2	Cefalexin	431
3	Cefazolin	413
4	Cefradine	330
5	Amoxicillin and beta-lactamase inhibitor	300
